# Mobile-Based Cognitive Behavioral Therapy for Health Care Workers’ Mental Health in Ecuador: Quasi-Experimental Study

**DOI:** 10.2196/58943

**Published:** 2025-08-05

**Authors:** Sandra Lorena Muñoz-Ortega, Rubén Vladimir Alvarado Muñoz, Daniela Santamaria Guayaquil, Jade Pluas-Borja, Marco Faytong-Haro

**Affiliations:** 1 Facultad de Ciencias de la Salud, Universidad Espiritu Santo, Km 2.5 vía a Samborondón, Samborondón, 092301, Ecuador, 593 0986510209; 2 Centro Interdisciplinario de estudios en Salud (CIESAL), Departamento de Salud Pública, Escuela de Medicina, Universidad de Valparaiso, Valparaiso, Chile

**Keywords:** mental health, depression, mobile apps, mental disorders, effectiveness, depressive symptoms, emotional distress, cognitive behavioral therapy, experimental study, design, Ecuador, psychological well-being, psychological, tool, health care worker, accessible, support system

## Abstract

**Background:**

Mental health challenges, including depression, anxiety, and burnout, have become increasingly prevalent among health care workers, who face high-stress environments, limited resources, and long working hours. The COVID-19 pandemic has intensified these issues, especially in regions like Latin America, where health care professionals experience heightened anxiety and depression. The urgent need for mental health support has prompted the development of mobile health (mHealth) solutions. These tools offer accessible, confidential interventions that help reduce stigma and encourage engagement. The “Psicovida” mobile app was designed to provide cognitive behavioral therapy (CBT)–based activities tailored to health care workers, supporting them in managing stress, anxiety, and depression.

**Objective:**

This study aims to evaluate the effectiveness of Psicovida, a mobile app that delivers CBT-based interventions, in reducing depressive symptoms and emotional distress among health care workers over a 3-month period.

**Methods:**

A quasi-experimental, nonrandomized controlled study was conducted with health care workers at a public hospital in Ecuador. Participants were recruited offline and assigned to either an intervention group that used the Psicovida app or a control group that received no intervention. The app provided weekly CBT-based tasks focused on stress management, cognitive restructuring, and emotional regulation. Data collection included demographic information, with mental health outcomes assessed pre- and postintervention using the Patient Health Questionnaire-9 (PHQ-9) to measure depression and the General Health Questionnaire-12 to assess overall psychological well-being.

**Results:**

A total of 211 health care workers participated, with 88 in the intervention group and 96 in the control group, and 29 participants dropped out. Among the intervention group, adherence varied: 34% (30/88) used the app consistently for 10‐12 weeks, 42% (37/88) for 7‐9 weeks, and 24% (21/88) for fewer than 6 weeks. Significant improvements in mental health outcomes were observed among app users. The intervention group exhibited a statistically significant reduction in depressive symptoms, with PHQ-9 scores decreasing significantly (*P*<.001; 95% CI 6.17-9.36). Within this group, 20% (18/88) achieved complete remission of depressive symptoms (PHQ-9 scores <5), 32% (28/88) showed mild symptoms (PHQ-9 scores=5‐9), and 48% (42/88) remained in the range requiring treatment referral (PHQ-9 scores ≥10). General Health Questionnaire-12 scores similarly showed substantial improvement in psychological well-being (*P*<.001; 95% CI 3.99-5.58).

**Conclusions:**

The Psicovida mobile app demonstrates promise as an accessible, effective tool for reducing depression and anxiety among health care workers through CBT-based interventions. This study highlights the potential of mHealth technology to deliver targeted mental health support, especially in resource-limited settings. Future research should focus on evaluating long-term impacts and broader applications in varied health care environments.

## Introduction

### Background

Mental health has emerged as a global challenge affecting millions of individuals across geographical and cultural boundaries. Mental disorders, including depression and anxiety, now affect over 450 million people globally, according to the World Health Organization, posing serious consequences for quality of life and productivity. Events like the COVID-19 pandemic have only exacerbated these challenges, highlighting the urgent need for innovative and accessible solutions [1].

Health care workers, especially doctors and nurses, are among those most affected by the mental health crisis, facing high rates of depression, anxiety, and burnout. Research shows that nearly half of junior doctors report symptoms of depression, while over 63% experience significant anxiety. Physicians also face burnout rates 20%‐30% higher than those of other professions and are 1.4 to 2.3 times more likely to die by suicide [2]. Despite these concerning statistics, many health care workers hesitate to seek mental health support due to stigma and fear of professional consequences [3]. This hesitation highlights the critical need for accessible, confidential mental health resources that encourage help-seeking within health care environments, fostering a culture where mental health support is accepted and valued.

In Latin America, health care professionals contend with additional mental health challenges, worsened by systemic issues like insufficient health care infrastructure, extended work hours, and direct exposure to the COVID-19 crisis. Anxiety affects between 10.6% and 76.5% of health care professionals, with depression impacting up to 81% in some regions. In Ecuador, for example, 46.4% of health care workers report experiencing anxiety, while 34.5% report depression. Among female obstetricians in Peru, over half exhibit depressive symptoms [4]. Frontline workers, particularly young and female health care professionals, face heightened vulnerability due to high workloads, inadequate personal protective equipment, and ethical dilemmas, leading to both physical and mental exhaustion [5]. The prevalence of these mental health issues points to an urgent need for and met the inclusion criteria effective psychological support and systemic improvements in working conditions.

Addressing these challenges requires immediate attention to systemic reforms that include accessible and confidential mental health services. In this context, mHealth solutions have emerged as promising tools. Recent studies have assessed the effectiveness of mHealth interventions targeting various mental health issues, including depression [6,7], suicide [8], schizophrenia [9], substance use disorders [10], and psychosis [11], among others [12]. Recent systematic reviews focused on assessing smartphone apps for mental health demonstrated their ability to generate significant reductions in anxiety [13] and depression [13]. mHealth apps have been proven particularly effective in breaking down barriers to help-seeking, such as stigma and time constraints, and are shown to improve user engagement when co-designed with health care workers [14]. Additionally, mHealth tracking apps have been recognized for increasing self-awareness and enabling users to manage their symptoms by identifying triggers and patterns. This kind of proactive self-management can be highly beneficial for health care workers facing depression and burnout [15]. These digital tools, if implemented widely, could play a pivotal role in addressing the ongoing mental health crisis among health care workers.

### Objective

This study aims to bridge the gap in understanding the effectiveness of mHealth interventions in mitigating mental health issues among health care workers. By conducting a quasi-experimental, nonrandomized controlled trial among health care workers in a public hospital in Ecuador, this research seeks to evaluate the impact of the “Psicovida” mobile app [16], a digital tool designed to support mental health through cognitive behavioral therapy (CBT)–based activities, on depression and emotional distress symptoms. The app offers structured interventions aimed at alleviating symptoms associated with mental health disorders, providing users with accessible and guided support. The unique challenges faced by health care personnel in Latin America, especially in regions like Ecuador, where systematic monitoring of mental health issues is lacking, underscore the importance of this research.

## Methods

### Study Design and Setting

This study used a quasi-experimental, nonrandom, parallel, controlled trial with pre- and postintervention assessments. It involved 2 groups with similar baseline characteristics: an intervention group receiving access to the Psicovida app and a control group without access. The study started in January 2023, coinciding with a surge in COVID-19 cases in Ecuador. This wave significantly impacted the health care system, creating heightened stress among health care workers [17]. The timing of the study underscores the critical need for effective mental health support mechanisms for this population.

The trial adheres to the Consolidated Standards of Reporting Trials (CONSORT) eHealth guidelines, and the detailed study protocol and statistical analysis plan are publicly available on ClinicalTrials.gov (NCT06650449) [18,19].

### Rationale for Quasi-Experimental Design

The decision to use a quasi-experimental approach was driven by the desire to achieve homogeneity in key demographic characteristics (age, gender, marital status, education level, and profession) between the intervention and control groups. To achieve this, participants were purposefully assigned to 2 cohorts: those who used the Psicovida app and those who did not. This intentional grouping aimed to control for potential confounding variables, acknowledging the limitation that true random assignment was not used.

### Participants

The study population comprised health care workers (doctors and nurses) from various departments of a public hospital in Quito, Ecuador. Participants were purposefully assigned to 2 groups—those who used the Psicovida app and those who did not—to control for potential confounding variables, acknowledging the limitation that true random assignment was not used. At the request of hospital administrators, some staff from high-acuity wards, whose heavy workloads left no additional time for training, were kept in the control group, whereas personnel from outpatient clinics with more flexible schedules were placed in the intervention group. Inclusion criteria were (1) employment as health care personnel at the designated hospital for at least 12 months, (2) ownership of a smartphone, and (3) provision of informed consent. Participants included both men and women aged 18 years and older, regardless of marital status. These criteria ensured engagement with the Psicovida app and effective completion of study tasks.

Individuals not meeting any of the above inclusion criteria were excluded from participation.

### Recruitment and Data Collection

Recruitment and data collection occurred primarily through the Psicovida app between October 2022 and January 2023, using 3 strategies. First, informational meetings were conducted by the research team to explain the study and the Psicovida app and to obtain digital consent. Second, from an initial pool of 296 health care professionals who expressed interest and met the inclusion criteria, 211 eligible participants were selected based on EPIDAT software (EPIDETAL, developed by the Epidemiology Service of the Dirección Xeral de Saúde Pública, Consellería de Sanidade (Xunta de Galicia), with support from the Pan American Health Organization (PAHO/WHO) and CES University of Colombia) recommendations and assigned to either the intervention or control group. A double-entry table was used during the assignment to ensure balanced distribution across key variables such as profession and sex. Finally, baseline data were collected, including personal information (email, age, gender, and education level) for participant profiling, and psychological evaluations using the Patient Health Questionnaire (PHQ-9) and the General Health Questionnaire (GHQ-12) to assess emotional distress and depressive symptoms.

During the study design phase, a plan was implemented to account for potential dropout rates, including strategies to manage missing data and maintain sample integrity. The study was conducted remotely via the Psicovida app, with minimal direct interaction with the research team, except when participants opted to initiate communication.

### Intervention

The Psicovida app is a free tool designed to support health care workers’ mental health by providing CBT-based activities aimed at alleviating symptoms of depression and emotional distress. The app includes a structured program of 12 weekly activities, each taking around 2 to 10 minutes to complete, focusing on introspection, stress management, and the restructuring of negative thoughts. The modules cover various aspects of mental health and personal growth, delivered through interactive tasks that address emotional, cognitive, and physical dimensions.

**Figure 1.**
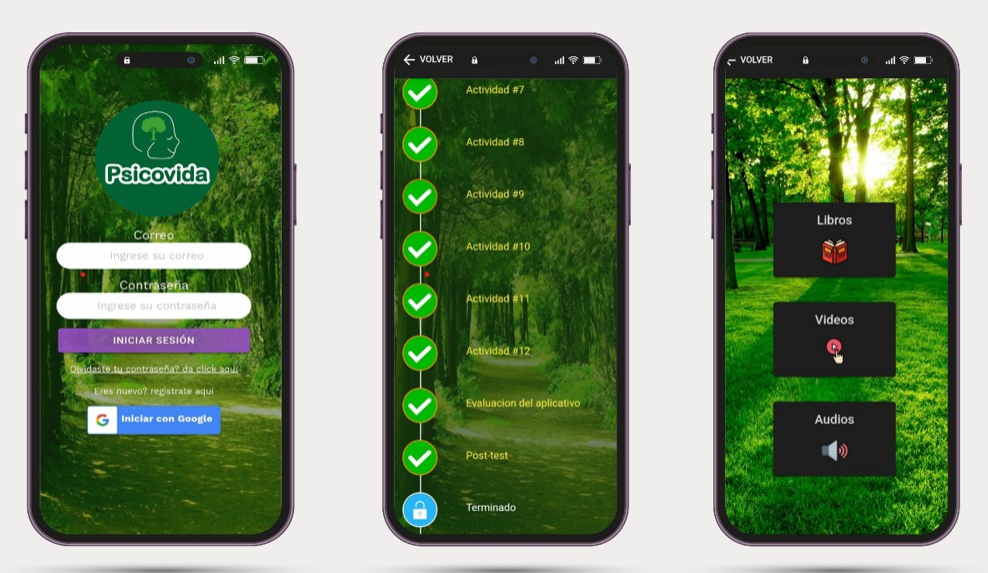
Psicovida mobile app.

The app was available to all interested users for download from the Google Play Store, without the requirement to belong to a specific group to access it. Participants did not pay for access, nor did they receive any financial compensation for their participation. During the onboarding phase, participants were encouraged to engage with the app regularly, completing the assigned activities over a period of up to 3 months. The app sent daily reminder emails to participants from Monday to Friday, encouraging consistent participation and improving user engagement. Users could adjust the settings for reminders, allowing flexibility in how they interact with the app. Users could adjust the settings for reminders, allowing flexibility in how they interact with the app. You can see the app interface in Figure 1.

Since its initial release, significant updates have been made to improve user experience, including expanding system bandwidth to address downtimes and enhancing the app’s user interface based on feedback from previous users. These changes, validated by clinical psychology experts, ensure that the therapeutic quality of the activities remains intact, offering personalized support to health care workers. Developed as Discord Psicovida Version 1 by principal author (SMO), the program underwent rigorous evaluations to align with the needs of its user base. For digital preservation and access, the Psicovida app can be accessed via a dedicated link on the Open Science Framework repository [20]. Upon completing the program, participants received a notification thanking them for their participation and were provided with general recommendations for maintaining long-term mental health. The description of each activity is presented in Multimedia Appendix 1.

At the end of the app use period, participants provided feedback through a series of structured questions. This feedback will help guide improvements for future versions of the app, based on user experiences and suggestions.

### Assessments

#### Depression

The PHQ-9 was carefully implemented as a digital assessment tool to ensure data reliability and participant engagement. Participants provided informed consent online after being fully informed about the purpose of the PHQ-9, confidentiality measures, and their data privacy rights. The questionnaire maintained its standard, validated format with nine items, each required to be answered to ensure complete data collection. The questions were presented in a sequential, user-friendly interface that preserved the questionnaire’s established structure without adaptive questioning or alterations.

Participant completion was monitored at both baseline and postintervention to assess adherence and track changes in depression severity over time. Unique identifiers were used to ensure that each participant completed the PHQ-9 only once at each time point, eliminating the risk of duplicate entries. Data security was prioritized through encryption and secure storage of responses. Although specific online validation was not conducted, the PHQ-9’s high internal consistency (Cronbach α>0.80) and proven sensitivity and specificity in various settings support its reliability for this digital deployment [21].

#### Anxiety

The GHQ-12 was implemented digitally to assess emotional distress while upholding rigorous standards for data quality and user experience. Before beginning the GHQ-12, participants reviewed and agreed to informed consent details, which clarified the study’s objectives, the role of the GHQ-12, and data protection practices. All 12 items were mandatory, ensuring comprehensive data for each participant and maintaining the GHQ-12’s validated structure. The questionnaire was presented in a fixed, linear format, allowing participants to complete it with ease and consistency, without adaptive questioning or alterations.

Completion rates were monitored both at the beginning and end of the study to evaluate participant engagement and the tool’s effectiveness in capturing changes in emotional distress. Each participant was assigned a unique identifier to track usage and prevent multiple entries, supporting data integrity. Responses were securely encrypted and stored, safeguarding participant confidentiality. While no digital-specific validation of the GHQ-12 was mentioned, its established reliability (Cronbach α ranging from 0.70 to 0.90) and consistent performance across studies reinforce its suitability for digital application [22].

### Demographic and Lifestyle Variables

Information on age, gender, marital status, education level, profession, and toxic habits (alcohol and tobacco consumption) was collected to describe the study population and adjust for potential confounders.

### Procedure

The Psicovida quasi-experimental trial followed a structured process to ensure participant engagement and data collection. After recruitment and eligibility confirmation in January 2023, participants were randomly assigned to either the intervention group, which used the Psicovida app, or the control group, which did not receive app access.

Both groups completed baseline assessments, including demographic data collection and psychological evaluations using validated tools (PHQ-9 for depression and GHQ-12 for emotional distress). Following this, the intervention group started app use, while the control group received standard mental health support information. Outcome assessments were conducted at two time points: (1) baseline (start of the study) and (2) study completion (end of 3 months). Participants received periodic email reminders to encourage adherence to the study protocol and completion of assessments. The intervention group was encouraged to engage with the app weekly for up to 12 weeks, with each activity designed to take 2‐10 minutes. Upon completing the study, participants had the option to continue using the app, subject to updates or maintenance periods.

The study ran from January 2023 to March 2023, spanning three months.

### Data Management and Monitoring

All study data were collected and recorded electronically through the Psicovida mobile app. The data, including identifiable information such as participants’ email addresses and demographic details, was stored in a secure, encrypted database managed by the research team. Identifiers were removed during data processing to ensure confidentiality before analysis. Weekly backups of all data were conducted by the research team during the data collection phase, and backups were securely stored in a password-protected environment. Only approved members of the research team and designated technical staff had access to the data.

### Trial Harms and Risk Management

Given the sensitive nature of mental health content, it was possible that some participants could experience distress while using the Psicovida app. Although no serious adverse events were anticipated based on previous trials of similar apps, the research team recorded and monitored any adverse events reported by participants. Adverse events were defined as any reported psychological distress or health impacts resulting from app use. The principal investigator documented and notified the ethics committee within 24 hours of any adverse events. If necessary, a data safety monitoring board consisting of independent clinical reviewers assessed the situation and recommended whether the study should continue. The app also provided direct access to mental health support services via a “reach out” button on the app’s home screen, allowing users to contact help if needed.

### Data Analysis

To determine the appropriate sample size for this study, EPIDAT, a specialized statistical software, was used with a one-sided hypothesis anticipating improved outcomes in the intervention group. Based on a 95% CI and a 5% margin of error, EPIDAT recommended reducing the initial pool of 296 health care personnel to a final sample size of 211 participants. Of these, 96 were allocated to the control group and 115 to the intervention group. However, there was an approximate loss of 29 participants, representing about 15% of the recommended sample size, due to personnel leaving the hospital or not completing the study.

As the study required active participant engagement with the Psicovida app, blinding participants to group allocation was not feasible. However, outcome assessors and data analysts were blinded to group assignments to reduce bias during data collection and analysis. Fortunately, there was no attrition during the study period, ensuring that all participants completed both baseline and postintervention assessments, thereby preserving the integrity of the data and the validity of the findings.

Descriptive statistics were used to summarize demographic characteristics, with chi-square tests for categorical variables and *t* tests (2-tailed) for continuous variables to compare distributions between groups. The impact of the Psicovida app on depression and emotional distress was analyzed using paired *t* tests for within-group comparisons and independent *t* tests to assess changes between groups. Effect size calculations were performed to evaluate the clinical significance of these findings.

To further understand the intervention’s impact, binary logistic regression models were constructed to examine predictors of depression (PHQ-9 scores) and emotional distress (GHQ-12 scores) at the end of the study, considering factors such as app use, baseline psychopathology, and demographic variables. All statistical analyses were conducted using SPSS software (IBM Corp), with significance set at *P*<.05, based on data sourced from the Psicovida app database as of March 2023.

### Ethical Considerations

This study received approval from the Human Research Ethics Committee of the Carlos Andrade Marín Hospital (registration number IESS-HCAM-CEISH-2022‐0007) on April 21, 2022. All procedures adhered to the ethical standards of the institutional and national research committee, as well as the principles of the Declaration of Helsinki. No exemptions from ethical review were sought, as the study involved human participants.

Participants provided informed consent digitally before enrolling in the study. The consent process included detailed information on the study’s purpose, objectives, potential benefits, risks, and confidentiality measures. Participants confirmed their voluntary agreement by checking a digital consent form. They were informed of their right to withdraw from the study at any time without repercussions.

To ensure participant privacy and confidentiality, all data collected through the Psicovida mobile app were anonymized prior to analysis. Identifiable information, such as email addresses, was encrypted and stored in a secure database accessible only to the research team. Unique identifiers were used to link participant data while preserving anonymity during analysis. No personally identifiable information was included in the final dataset.

Participants were not provided with financial or material compensation for their participation in this study. Instead, they were thanked for their contribution to advancing mental health research and given general recommendations for maintaining long-term mental health upon completing the study.

## Results

### Overview

**Figure 2.**
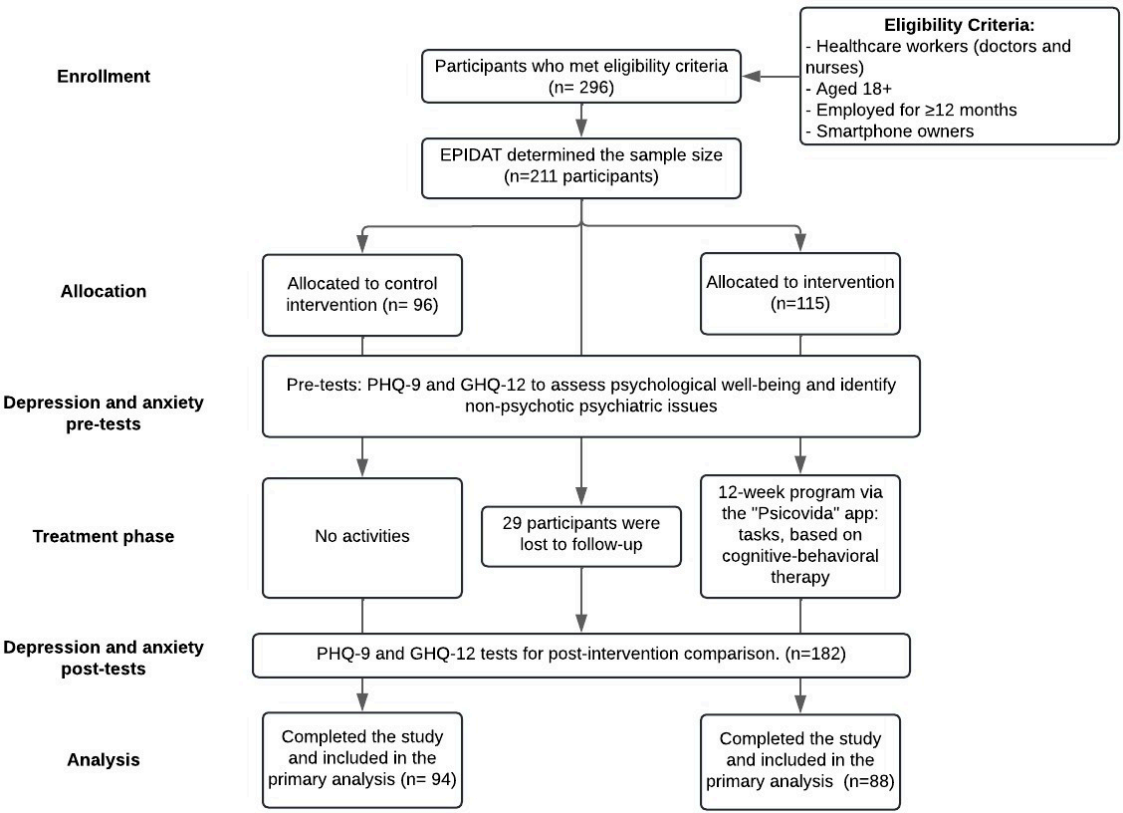
Psicovida study participation flowchart based on the CONSORT (Consolidated Standards of Reporting Trials) 2011 statement recommendations. GHQ-12: General Health Questionnaire-12; PHQ-9: Patient Health Questionnaire-9.

Between January 2023 and March 2023, 296 health care workers expressed interest in participating in the Psicovida study. Based on EPIDAT’s recommendations, the sample size was reduced to 211 eligible participants who provided informed consent and were assigned to either the Psicovida intervention group (n=115) or the control group (n=96). However, due to a 15% dropout rate, approximately 29 participants discontinued their involvement. As a result, the final distribution included 94 participants in the control group and 88 in the intervention group, who were evaluated for the primary outcome. The procedure for the Psicovida quasi-experimental trial is presented in Figure 2.

In terms of app use, 34% (30 participants) in the intervention group used the app for the full duration of 10‐12 weeks, 42% (37 participants) used it for 7‐9 weeks, and 24% (21 participants) used it for fewer than 6 weeks. This variation in usage was taken into account in the analysis to understand the impact of exposure levels on primary outcomes.

Table 1 reveals a distinct age group distribution within the app use group, primarily due to the higher percentage of individuals aged between 18 and 25 years. Both groups exhibited a uniform distribution of sex and marital status, with females and single individuals prevailing. Similarly, the predominant education level was undergraduate studies, consistent with the most common age group. As for profession, despite having inversely proportional frequencies in the study groups, there was no statistically significant difference. Overall, less than 11% (n=19) of the studied participants engaged in alcohol or tobacco consumption.

**Table 1.** Distribution of demographic variables in the study groups.^a^

Demographic variables	Intervention group		Group without intervention		*P* value
Age (years), n (%)					<.001
18‐25	54 (61.4)		46 (48.9)		
26‐35	10 (11.4)		18 (19.2)		
36‐55	20 (22.7)		20 (21.3)		
Over 55	4 (4.5)		10 (10.6)		
Sex, n (%)					.13
Female	60 (68.2)		54 (57.4)		
Male	28 (31.8)		40 (42.6)		
Marital status, n (%)					.23
Single	43 (48.9)		46 (48.9)		
Divorced	8 (9.1)		12 (12.8)		
Free union	19 (21.6)		13 (13.8)		
Married	18 (20.4)		23 (24.5)		
Educational level, n (%)					.38
Undergraduate	77 (87.5)		86 (91.5)		
Postgraduate	11 (12.5)		8 (8.5)		
Profession, n (%)					.11
Doctors	48 (54.5)		40 (42.6)		
Nurses	40 (45.5)		54 (57.4)		
Toxic habits, n (%)					
Alcohol consumption	15 (17.0)		4 (4.3)		.005
Tobacco consumption	5 (5.7)		19 (20.2)		.004

a Source: Database of the Psicovida mobile app, March 2023.

### Evaluation of Depression Symptoms at the Beginning and End of the Groups With and Without Use of the App (With the PHQ-9 Test)

Table 2 shows the scores in both groups at the start and end of the research. We categorized the PHQ-9 variable to discern the proportion of subjects with associated psychopathology according to the applied test evaluation.

The group using the app showed significant improvements (*P*<.001), with a 95% CI, with a difference of 6.17‐9.36. This suggests that in a sample similar to the one studied, the application of the app is anticipated to result in a decrease in the PHQ-9 test score from 6 to 9 points with 95% CI.

At the end of the application, 20% (18/88) of the participants met the criteria for complete remission or no symptoms of depression (PHQ-9 scores <5), 32% (28/88) met the criteria for recovery or mild symptoms (PHQ-9 scores=5‐9) and 48% (42/88) continued to meet the referral criteria for treatment (PHQ-9 ≥10). In the group that did not use the app, although a significant improvement was observed in some participants and there was a significant decrease in the mean in the final measurement (*P*=.03), the percentage that met the referral criteria for treatment (PHQ-9 scores ≥10) increased by 4%.

**Table 2.** Comparison of the results of the PHQ-9^a^ test in the study groups at the beginning and end of the research.^b^

	Start	End	*P* value
PHQ-9, mean (SD)			
Cluster with use of the app	16.77 (7.37)	9.00 (4.88)	<.001
Group without use of the app	16.13 (−7.1)	14.62 (5.48)	.03
PHQ-9 ≥10, n (%)			
Cluster with use of the app	78 (89)	42 (48)	<.001
Group without use of the app	79 (84)	83 (88)	.34

a PHQ-9: Patient Health Questionnaire-9.

b Source: Database of the Psicovida mobile app, March 2023.

**Figure 3.**
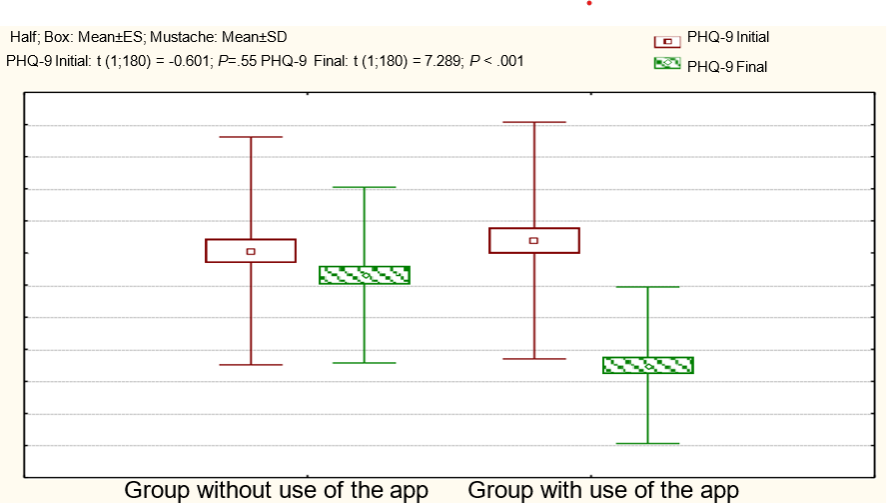
Comparison of the results of the PHQ-9 test in the 2 study groups. ES: estimated standard error; PHQ-9: Patient Health Questionnaire-9.

The following graph (Figure 3) shows the differences between the groups. The initial scores on the PHQ-9 test showed no statistically significant differences between the groups, but significant disparities emerged in the final scores. In the app user group, a decrease in the average was observed, and a large effect size was calculated (d=0.95).

### Evaluation of the Symptoms of Emotional Distress at the Beginning and End of the Research Groups (With the GHQ-12 Test)

Similarly, for the GHQ-12 test (Table 3), we see significant improvements in the group using the app (*P*<.001), with a 95% CI for the difference of 3.99‐5.58. This means that in a similar sample, using the app is expected to result in an average decrease in the GHQ-12 test score between 4 and 5 points, about 95% of the time.

**Table 3.** Comparison of the results of the GHQ-12^a^ test in the study groups at the beginning and end of the research.^b^

	Start	End	*P* value
GHQ-12 , mean (SD)			
Cluster with use of the app	14.53 (7.22)	9.75 (5.24)	<.001
Group without use of the app	15.15 (5.95)	17.04 (5.57)	<.001
GHQ-12 ≥16, n (%)			
Group with use of the app	55 (57.29)	10 (11)	<.001
Group without use of the app	41 (44.71)	54 (57)	.008

a GHQ-12: General Health Questionnaire-12.

b Source: Database of the Psicovida mobile app, March 2023.

In this group, 89% (78/88) of the participants met the criteria for complete remission or showed no distress symptoms (GHQ-12 scores <16), and only 11% (10/94) still met the referral criteria for treatment (GHQ-12 scores ≥16). Meanwhile, for the group without app use, the percentage of participants with psychopathology increased, and this difference became statistically significant compared to the initial proportion.

**Figure 4.**
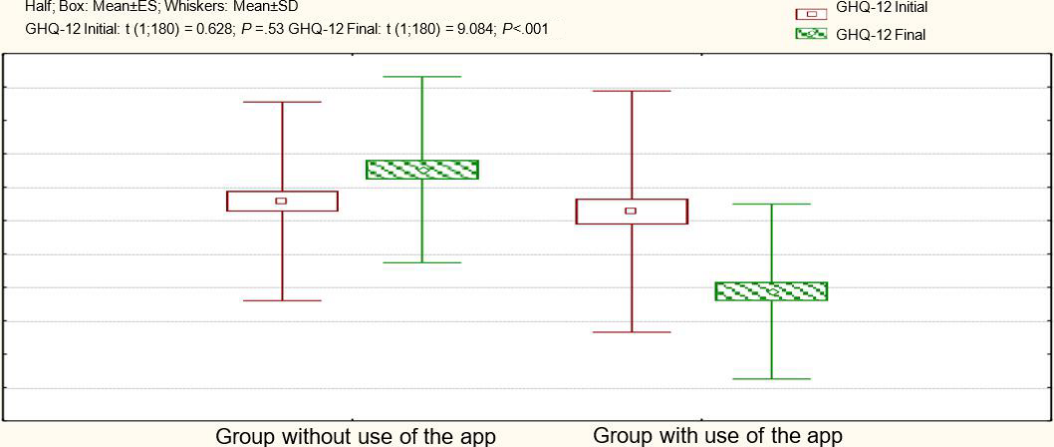
Comparison of the results of the GHQ-12 test in the 2 study groups. ES: estimated standard error; GHQ-12: General Health Questionnaire-12.

Figure 4 shows the GHQ-12 test results. Initially, both groups had similar scores. Towards the end, there are significant differences favoring the app users, with a noticeable effect (*d*=1.12). The group without app use also exhibits an increase in final scores.

### Binary Logistic Regression of the PHQ-9 Test

The PHQ-9 results in our study were dichotomized to categorize participants into 2 distinct groups based on their depression severity levels. Individuals scoring 10 or above were classified as having moderate to severe depressive symptoms, indicating a need for further clinical evaluation. Those with scores below 10 were considered to have minimal to mild depressive symptoms, suggesting lower levels of depression. This dichotomization allowed for a clearer analysis of the relationship between demographic factors such as sex, age group, marital status, educational level, or profession, and depression severity. Despite this categorization, our findings revealed no significant relationships between these demographic variables and the final PHQ-9 results.

The model (Table 4) had the presence of psychopathologies at the end of the research (PHQ-9*f*≥10) as the dependent variable. It includes 2 independent variables: the use of the Psicovida app (with the use of the app) and the variable (PHQ-9i≥10) representing the presence of psychopathologies at the beginning of the investigation, determined by the PHQ-9 test itself. This model shows a significance of less than .05 in the omnibus test, indicating that it contributes to explaining the observed event.

**Table 4.** Variables in the equation, PHQ-9^a^. Variables introduced in step 1: with use of the app and PHQ-9i≥10.

Variables	b	SE	Wald chi-square (*df*)	*P* value	Odds ratio (95% CI)
With use of the app^b^	−2.269	0.398	32.513 (1)	<.001	0.103 (0.047-0.226)
Step 1a PHQ-9i≥10^c^	2.196	0.498	19.458 (1)	<.001	8.987 (3.388-23.841)
Constant^d^	−0.166	0.419	0.157 (1)	.69	0.847 (N/A)^e^

a PHQ-9: Patient Health Questionnaire-9.

b With use of the app denotes a binary indicator that captures whether participants engaged with the mobile app during the intervention period (coded 1 for use, 0 for nonuse).

c Step 1a PHQ-9≥10 is a baseline severity flag identifying individuals whose initial PHQ-9 score met or exceeded the clinical threshold of 10, indicating moderate-to-severe depressive symptoms (coded 1 for ≥10, 0 otherwise).

d Constant represents the model intercept—the log-odds of screening positive when both predictors are set to zero (that is, for nonapp users with a baseline PHQ-9 <10).

e N/A: not available.

The independent variables of the model explain up to 37% of the dependent variable, and the correct classification level of the model is 72%. The first independent variable, the use of the Psicovida app, has a negative coefficient, an odds ratio (OR) <1, and therefore represents a protective factor, contrary to what happens with the initial presence of psychopathologies, which shows a positive coefficient, an OR>1; therefore, it is considered a risk factor.

### Binary Logistic Regression of the GHQ-12 Test

There were no significant relationships between sex, age group, marital status, educational level, or profession in the final GHQ-12 results. As well, no association was observed between the initial or final values obtained in the PHQ-9 test (Table 5).

**Table 5.** Variables in the equation, GHQ-12^a^. Variables introduced in step 1: with use of the app and GHQ-12i≥16.

Variables	b	SE	Wald chi-square (*df*)	*P* value	Odds ratio (95% CI)
With use of the app^b^	−3.859	0.612	39.697 (1)	<.001	0.021 (0.006-0.070)
Step 1a GHQ-12i≥16^c^	3.093	0.588	27.695 (1)	<.001	22.054 (6.968-69.798)
Constant^d^	−0.778	0.292	7.085 (1)	.008	0.459 (N/A)^e^

a GHQ-12: General Health Questionnaire-12

b With use of the app denotes a binary indicator that captures whether participants engaged with the mobile app during the intervention period (coded 1 for use, 0 for nonuse).

c Step 1a GHQ-12i≥16 is a baseline severity flag identifying individuals whose initial GHQ-12 score met or exceeded the clinical threshold of 16, indicating moderate-to-severe anxiety symptoms (coded 1 for ≥16, 0 otherwise).

d Constant represents the model intercept—the log-odds of screening positive when both predictors are set to zero (that is, for nonapp users with a baseline GHQ-12 <16).

e N/A: not available.

The model used a dependent variable representing the presence of psychopathologies at the conclusion of the study (GHQ-12*f*≥16), and as independent variables, the use of the Psicovida app and the variable (GHQ-12i≥16), which represents the presence of psychopathologies at the beginning of the research (determined by the GHQ-12 test itself). The model demonstrates a *P* value below .05 in the omnibus test, suggesting it significantly contributes to understanding the event in question.

The independent variables of the model explain up to 54% of the dependent variable, and the correct classification level of the model is 83%. The first independent variable, the use of Psicovida application, has a negative coefficient, an OR <1, and therefore represents a protective factor, contrary to what happens with the initial presence of psychopathologies, which shows a positive coefficient, an OR >1; therefore, it is considered a risk factor.

## Discussion

### Principal Findings

In this study, we evaluated the effectiveness of Psicovida, a mobile app–based CBT intervention, in reducing depressive symptoms and emotional distress among health care workers. The results demonstrated its efficacy as a mental health treatment option, with high user engagement, positive feedback, and significant reductions in depression severity—nearly half of the participants showed improvement, and 20% achieved full remission after three months. These findings, comparable to those from face-to-face CBT, indicate that Psicovida could be a scalable solution in resource-limited settings.

Moreover, the data revealed that demographic factors such as age, sex, education, profession, and marital status did not significantly influence the intervention’s success, particularly in urban areas like Quito. This suggests that mHealth applications like Psicovida can be effective across diverse user profiles, making it a versatile tool for addressing mental health challenges in health care workers.

### Perspectives

A pivotal aspect of this investigation centers on the efficacy of Psicovida in ameliorating symptoms of anxiety and depression. The initial findings indicated that 16.77% of app users reported persistent symptoms of anxiety and depression, a figure that notably declined to 9% following the app’s use. This contrasted with the control group, which exhibited a negligible shift in symptomatology, underscoring the app’s potential therapeutic value. This outcome resonates with the results observed in other mHealth interventions, such as the MHapp app, which amalgamates three components (Moodkit, MoodPrism, and MoodMission) rooted in CBT principles, achieving similar reductions in anxiety and depression symptoms [23].

Similarly, the Mindset for Depression app, which combines structured cognitive behavioral tasks with therapist guidance, has demonstrated comparable reductions in depression severity [24]. In contrast, user-driven apps focusing on self-management and flexible health-tracking features, as highlighted in another study, show the importance of personalization in enhancing user engagement and outcomes. These parallels further underscore Psicovida’s potential in reducing depression and emotional distress, even without direct therapist involvement, demonstrating the scalability and accessibility of its design in supporting mental health among health care workers.

Intellicare, another app developed with Intervention Technology and incorporating diverse media such as videos, audio files, and animations [25], mirrors Psicovida’s approach in behavior and cognition modification. A distinctive featu*re* of Intellicare is its provision of brief individualized assistance by professionals, a facet absent in Psicovida. Despite this difference, Intellicare’s impact on reducing anxious and depressive symptoms, as assessed by the PHQ-9 and Generalized Anxiety Disorder-7 questionnaires, showcases significant parallels with Psicovida, reinforcing the potential of varied mHealth designs to effectively address mental health challenges.

The discourse extends beyond symptom reduction to the enhancement of positive psychological attributes, as evidenced by apps like OneUS and My Coping Plan [26]. These apps aim to cultivate positive emotions and thoughts, thereby elevating general well-being. The OneUS app, for instance, demonstrated a significant uplift in psychological well-being among its users, suggesting that mHealth interventions can play a crucial role in not only mitigating mental health symptoms but also in promoting positive mental states. This dual capability underscores the transformative potential of mHealth solutions in both therapeutic and preventive mental health contexts [27].

Writing exercises have long been recognized as a valuable tool in digital therapeutic interventions for their ability to foster self-reflection and cognitive restructuring. In Psicovida, these activities serve as a core component, encouraging users to explore their thoughts and emotions in a structured manner to facilitate changes in thinking patterns [28]. Compared to apps like Wysa, which use artificial intelligence (AI) for real-time, adaptive responses to users’ concerns, Psicovida emphasizes a guided and introspective process. However, integrating AI into Psicovida could enhance interactivity by offering personalized feedback or tailored guidance during writing tasks. This hybrid model would merge the depth of structured exercises with the adaptability of AI, potentially increasing user engagement and broadening its appeal [29].

Despite the encouraging trajectories observed in Psicovida and similar mHealth apps [30], the research landscape in Latin America, and indeed globally, reveals a pronounced scarcity in scientifically rigorous studies validating the clinical efficacy of these digital interventions. The prevailing emphasis on app usability and feasibility studies, while critical, underscores a nascent field that is yet to fully embrace the rigorous evaluation of clinical effectiveness [31]. This gap highlights an urgent need for comprehensive, long-term studies that can provide a robust evidence base to inform the integration of mHealth solutions into standardized mental health care practices, especially in Latin America [32].

Psicovida’s adaptability is further demonstrated by its potential to serve diverse user groups. For instance, adaptations inspired by Stay Strong, an app used to support the emotional well-being of Australian Indigenous groups [33], could make Psicovida effective in addressing stress and anxiety in incarcerated populations. Similarly, features from Intellect, which aids medical students in managing subclinical obsessive-compulsive disorder symptoms [34], could be incorporated to support those facing high stress levels. In the aftermath of global crises, such as the COVID-19 pandemic, Psicovida could adopt elements from apps like Foundations to provide structured and accessible mental health support [35].

The success of mHealth apps like Psicovida, however, depends on addressing both patient needs and mental health professionals’ recommendations. Psychiatrists often cite barriers such as a lack of individualization, inadequate data privacy protections, and the perception of these tools as supplements rather than replacements for in-person therapy, especially in complex cases [36]. Psicovida addresses many of these concerns by using CBT principles, offering an intuitive design, personalized content, and flexible scheduling, which optimize user experiences and complement traditional treatments. Nevertheless, maximizing the impact of such tools requires respecting the therapeutic relationship and reinforcing clinical work, ensuring their acceptance by both patients and professionals [37].

In synthesizing these insights, it becomes evident that mHealth applications like Psicovida hold considerable promise in transforming mental health interventions. Their capacity to transcend traditional demographic limitations, coupled with their efficacy in reducing mental health symptoms and enhancing positive psychological well-being, positions them as valuable assets in the mental health care continuum [38-41]. However, the journey towards their full integration into mainstream mental health services necessitates a concerted effort to bolster the scientific rigor of efficacy studies, ensuring these digital tools can be deployed with confidence in clinical and preventive mental health settings. As this field continues to evolve [42-44], the intersection of technology and psychology heralds a new era in mental health care, characterized by accessibility, personalization, and a holistic approach to well-being.

### Limitations

Despite the significant improvements observed in mental health outcomes for health care workers using the ’Psicovida’ mobile app, it is important to acknowledge and critically assess several limitations inherent to these findings, aligning with the best guidelines for eHealth practices [37]. First, the quasi-experimental design presents limitations. While the study ensured comparable characteristics between groups, a fully randomized controlled trial (RCT) would enhance internal validity by further reducing the likelihood of confounding variables influencing outcomes.

Second, this study was conducted in a single public hospital in Quito, Ecuador, which may limit the generalizability of the findings to other health care settings or regions. Factors such as organizational culture, health care policies, and available resources vary across institutions and countries, potentially impacting the app’s effectiveness in other contexts. Expanding future studies to include multiple institutions in diverse geographic locations would provide more robust evidence of the app’s applicability.

Third, the study intervention lasted only 3 months, making it challenging to assess the long-term sustainability of the app’s impact on mental health outcomes. Mental health interventions often require ongoing support to maintain improvements, and it remains unclear whether participants’ mental health would regress postintervention without continued app use or alternative support mechanisms. Additionally, participant engagement with the app varied, with 34% of participants using it for 10‐12 weeks, 42% (n=37) for 7‐9 weeks, and 24% (n=21) for fewer than 6 weeks. However, the study did not explore how these variations in engagement levels might have influenced outcomes. Understanding the relationship between frequency and intensity of app use and mental health improvements would offer valuable insights into optimal usage patterns.

Another limitation involves the reliance on self-reported measures, such as the PHQ-9 and GHQ-12, which may introduce bias as participants could underreport or overreport symptoms due to social desirability or personal perception. While these instruments are widely validated, incorporating objective measures of mental health, like clinical interviews or physiological data (eg, sleep patterns or stress biomarkers), could provide more comprehensive and unbiased assessments.

Finally, typical limitations in eHealth trials apply here, including the lack of blinding for participants, which could introduce expectation bias. Additionally, the multiplicity of outcomes assessed increases the risk of type I errors. Privacy concerns and technical issues, though minimal, were not fully explored in this study but should be considered in future research, as unintended effects in eHealth interventions may also include both negative and positive incidental findings.

In this non-RCT, structured reminders and prompts were used to encourage regular app engagement, which may have positively influenced adherence and outcomes. However, in a routine application setting, these elements may be absent, potentially impacting user adoption and consistent usage. Additionally, the level of technical support and structured monitoring available during the trial might not be feasible in typical usage scenarios. The absence of these supportive features in everyday settings could lead to decreased engagement, highlighting the need for integrating alternative engagement strategies, such as in-app notifications or occasional follow-up messages, to maintain effectiveness outside the structured environment of a clinical trial.

### Generalizability

The findings of this trial have implications for similar health care settings but may have limited generalizability to broader populations. Given that this study focused specifically on health care workers, the results may not directly apply to a general internet population or patients outside of the study setting. Health care workers face unique stressors and mental health challenges, which may not be reflective of other groups, limiting the applicability of the intervention’s effects in different contexts.

Moreover, certain elements of the non-RCT, such as periodic reminders and structured monitoring, may have positively influenced user engagement and adherence. In a routine application setting, the absence of these supportive elements could reduce both app usage and its effectiveness. To improve generalizability, future implementations should consider integrating support features, such as reminders or optional human guidance, which may enhance engagement and outcomes outside of a controlled trial environment. Such adaptations could facilitate the adoption and sustained use of eHealth interventions, making them more practical and effective in broader health care and community contexts.

### Further Research

Future research should address these limitations to strengthen the evidence supporting the Psicovida app and similar mHealth interventions. A fully RCT design could enhance internal validity by minimizing biases that may arise from a quasi-experimental approach. Expanding the study across multiple health care institutions and regions, particularly in Latin America, is essential. The lack of robust mental health research in this region, especially among health care workers, underscores the need for broader evaluations to assess the app’s effectiveness in diverse and underrepresented contexts.

As this study concluded, Psicovida serves primarily as an adjuvant to traditional mental health treatments rather than a standalone solution. Future research should explore how app-based interventions like Psicovida can be integrated with conventional therapeutic approaches to maximize effectiveness. This is particularly relevant for individuals with more severe mental health conditions, where professional support and structured treatments are indispensable.

Longitudinal studies are needed to evaluate the long-term effects of the Psicovida app. While this study demonstrated promising short-term outcomes, further investigation is required to determine whether the observed mental health improvements persist over time and to identify the optimal frequency or duration of app use. These studies should also explore the potential need for supplementary interventions to sustain benefits, ensuring the app remains effective as a supportive resource.

Comparative studies between Psicovida, other mHealth apps, and alternative therapeutic interventions would provide valuable insights into best practices for app design and implementation. Such research could help refine strategies to address the unique challenges faced by health care workers and adapt interventions to specific cultural or occupational contexts.

Additionally, future research should examine the app’s effectiveness across various health care roles (eg, doctors, nurses, and administrative staff) and investigate the influence of workplace stressors or social support. Understanding how user engagement impacts mental health outcomes is critical, as it can inform strategies to optimize adherence and enhance effectiveness. Incorporating objective mental health assessments, such as clinical interviews or physiological measures, alongside self-reported outcomes would further bolster the evidence base.

By addressing these research gaps, future studies can help refine and expand the application of Psicovida and similar tools, making them more practical and effective in broader health care and community contexts.

### Conclusions

The Psicovida app represents a significant advancement in the accessibility and convenience of mental health resources, offering a valuable tool for users to access support anytime and anywhere. This is particularly beneficial for individuals facing barriers to traditional mental health services due to geographic, social, or economic constraints. The app incorporates a variety of therapeutic approaches, including CBT and mindfulness, and provides interactive tools for emotional support, therapeutic guidance, and progress monitoring.

Despite its benefits, Psicovida should not be seen as a replacement for face-to-face therapy, but rather as a complementary resource. The effectiveness of the app can vary based on the user’s specific condition, the app’s design quality, and the user’s engagement. Therefore, it’s crucial to have realistic expectations based on scientific evidence and to view such apps as part of a broader mental health strategy.

## Supplementary material

10.2196/58943 Multimedia Appendix 1 Additional figures and tables.

## Abbreviations

AI: artificial intelligence
CBT: cognitive behavioral therapy
CONSORT: Consolidated Standards of Reporting Trials
GHQ-12: General Health Questionnaire-12
OR: odds ratio
PHQ-9: Patient Health Questionnaire-9
RCT: randomized controlled trial
