# Supplementary material for: Mobile-Based Cognitive Behavioral Therapy for Health Care Workers’ Mental Health in Ecuador: Quasi-Experimental Study
Source: JMIR Hum Factors. 2025 Aug 5;12:e58943. doi: 10.2196/58943 (PMC12324899; doi:10.2196/58943)
Supplement: Multimedia Appendix 1 [file humanfactors-v12-e58943-s001.docx]

**Table S1.**

| **Number of activities** | **Description** |
| --- | --- |
| **Activity 1:** This activity involves identifying the emotions and feelings that the user is experiencing on that day, aiming to establish a mechanism for introspection. | How are you feeling today? Identify your emotions and feelings through the images displayed on the screen. Then, write a brief reflection on this feeling, indicating whether you think this emotional state is beneficial for your mental health, and create your own plan to improve your mood. |
| **Activity 2:** Self-image reflection. Participants will be asked to upload an image with which they identify (a historical figure, artist, thinker, etc.). They will also need to provide a written explanation of why they chose that image and how it relates to them. | Reflection on self-image. Participants will be asked to upload an image with which they identify (historical figure, artist, thinker, etc.) and write about why they chose that image and how it relates to them. |
| **Activity 3**: This activity is designed to help the user set short-term, medium-term, and long-term goals with the aim of maintaining a consistent life plan. | What are your life goals? Participants should answer questions across several areas in an agenda, detailing their personal, work, and academic goals, each aimed at improving their attitudes and skills in managing daily life. Setting deadlines for each goal and reviewing them weekly to track progress is recommended. |
| **Activity 4:** The Jacobson Progressive Relaxation technique is provided to equip users with tools for managing stress and anxiety. | Jacobson’s progressive relaxation. Through an instructional video, deep muscle relaxation (DMR) will be practiced throughout the week. This technique is recommended for its ease of learning, accessibility during moments of anxiety, and proven scientific value. It is effective for managing excessive tension in situations of anxiety, pain, anger, or depression.  “<https://youtu.be/m5DAzMq_ls0?si=qQmzlu5dRpHcdC6M>” |
| **Activity 5:** This activity provides the resource of physical activity, as it helps individuals relax and maintain mental balance by connecting with their own bodies. | Exercise during your free time is recommended. A video will show simple exercises that can be done at home or at work to improve your mood. Participants are encouraged to increase the frequency and duration of the exercises. Practice at least three times per week.  “<https://youtu.be/diFjQVUL7wk?si=hoX3IHLn3WXf3LXf>” |
| **Activity 6:** Coping with anxiety through counterconditioning, which involves eliciting a new response in place of anxiety in feared situations. | Anxiety management through exposure-based counterconditioning. This involves imagining anxiety-provoking situations and holding them in mind until the feeling changes, a technique known as systematic desensitization. Begin with situations that produce mild anxiety and pair them with relaxation. Practice at least once every two weeks. |
| **Activity 7:** Users are provided with simple reading material that emphasizes the importance of the roles we all play wherever we are, highlighting the significance and purpose of their role within the society in which they find themselves. | Read and comment if you have ever experienced a similar situation.  ““Once upon a time, in a place that could be anywhere, and at a time that could be any time, there was a beautiful garden filled with apple trees, orange trees, pear trees, and lovely rose bushes, all of them happy and content. Everything in the garden was joyful, except for one tree that was deeply sad. The poor tree had a problem: it didn’t know who it was. ‘What you need is focus,’ said the apple tree.  ‘If you really try, you could grow tasty apples. See how easy it is?’  ‘Don’t listen to him,’ insisted the rose bush. ‘It’s easier to grow roses. See how beautiful they are?’  The tree, in desperation, tried everything they suggested, but because it couldn’t be like the others, it felt increasingly frustrated.  One day, an owl—the wisest of birds—came to the garden, and seeing the tree’s despair, exclaimed:  ‘Don’t worry; your problem isn’t that serious. It’s the same as that of many beings on Earth. I’ll give you the solution: don’t dedicate your life to being what others want you to be… Be yourself, get to know yourself, and to do that, listen to your inner voice.’ And with that, the owl disappeared.  ‘My inner voice…? Be myself…? Get to know myself…?’ the tree wondered, when suddenly, it understood…  Closing its eyes and ears, it opened its heart, and at last, it could hear its inner voice saying:  ‘You’ll never grow apples because you’re not an apple tree, nor will you bloom every spring because you’re not a rose bush. You’re an oak tree, and your destiny is to grow big and majestic, to shelter birds, give shade to travelers, and add beauty to the landscape… You have a mission: fulfill it.’  The tree felt strong and confident in itself and set out to become everything it was meant to be.  Soon, it filled its space and was admired and respected by all. And only then was the garden completely happy.  I wonder as I look around…  – How many oaks don’t allow themselves to grow?  – How many rose bushes, out of fear, only grow thorns?  – How many orange trees don’t know how to bloom?  In life, we all have a destiny to fulfill, a space and a special plan from God.” |
| **Activity 8:** Self-verbalization control. During the week, participants will identify the negative words they use in anxiety-inducing situations. The aim is to analyze these words to assess their validity and usefulness. The goal is to replace these negative self-verbalizations with positive ones. | Self-talk control. Over the week, identify the negative words you use during anxious situations, analyze them, and replace them with positive and rational phrases. Write down negative words and replace them one by one in the table below. |
| **Activity 9:** Mood-boosting activities. During this week, participants should engage in one or more activities that bring them pleasure, based on their personal preferences. They should record these activities in their daily agenda. | Engage in activities that improve your mood this week and record them in your daily agenda. |
| **Activity 10:** During this week, participants should review the short-term, medium-term, and long-term goals established in Activity 3, with the aim of confirming or reconsidering them. | Review the short-, medium-, and long-term goals you set in Activity 3, with the aim of confirming or adjusting them. |
| **Activity 11:** Thought stopping. This technique is aimed at halting negative thoughts that may lead to emotional disturbances and anxiety, which hinder problem-solving rather than facilitating it. | Thought-stopping technique. This technique aims to stop negative thoughts that cause anxiety and replace them with positive ones to improve distress management. Participants are encouraged to apply this technique throughout the week.  “**Emergency contact:** The app will have alert sensors in case of any mental health risk, monitored through the words participants write in their tasks, as well as a 24/7 emergency line if they need assistance with any mental health issue.” |
| **Activity 12:** During this week, participants will conduct an analysis of their engagement with the mobile application. They are required to write a brief statement in no more than 60 words about how they believe the app has benefited them or not. | At the end of the week, write in no more than 60 words about how you believe participating in this app has benefited you or not. |

**Figure S1**: Application Interface

*Login*


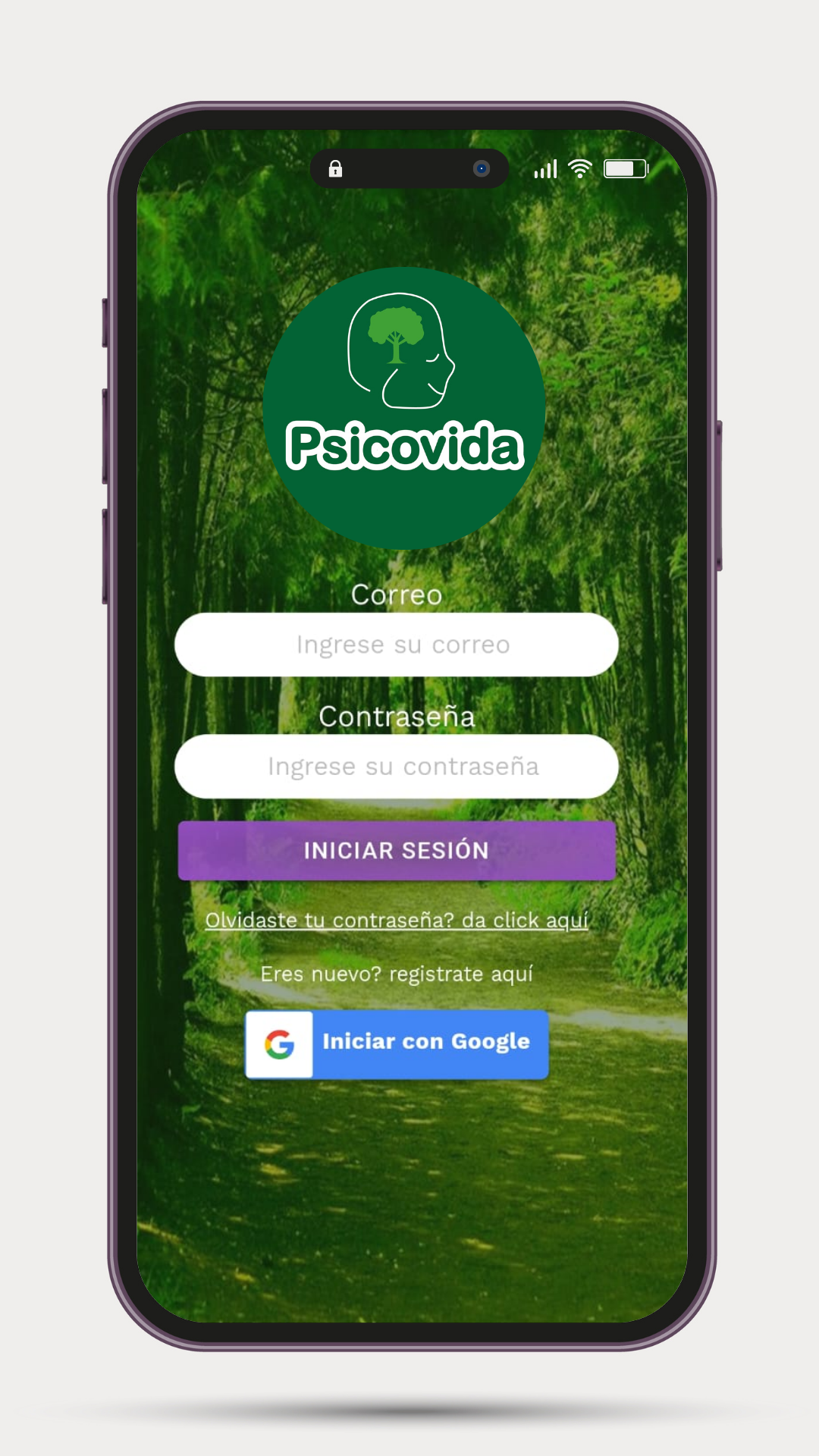


**Figure S2.**

*Main Menu*


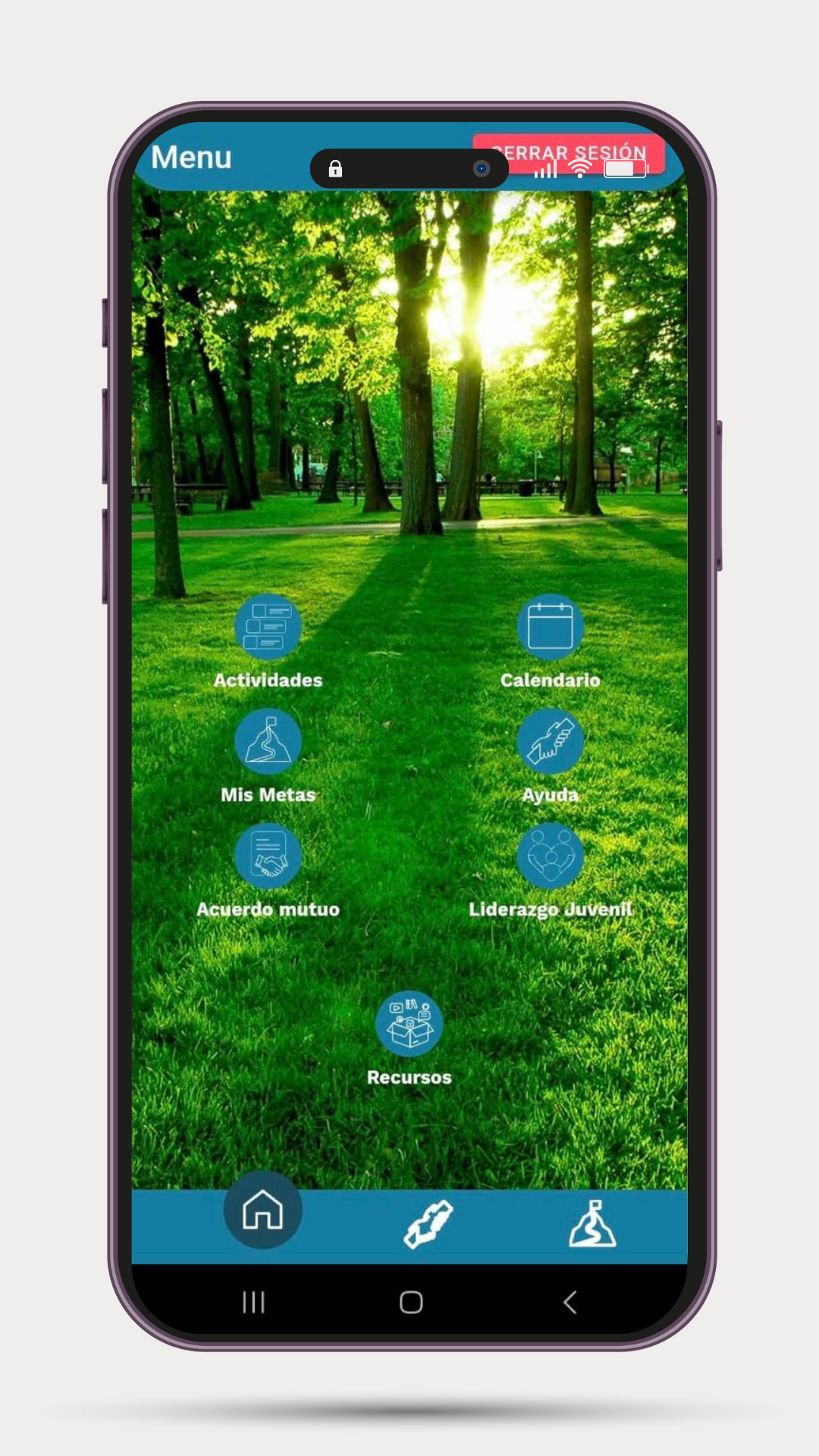


**Figure S3.**

*List of activities*


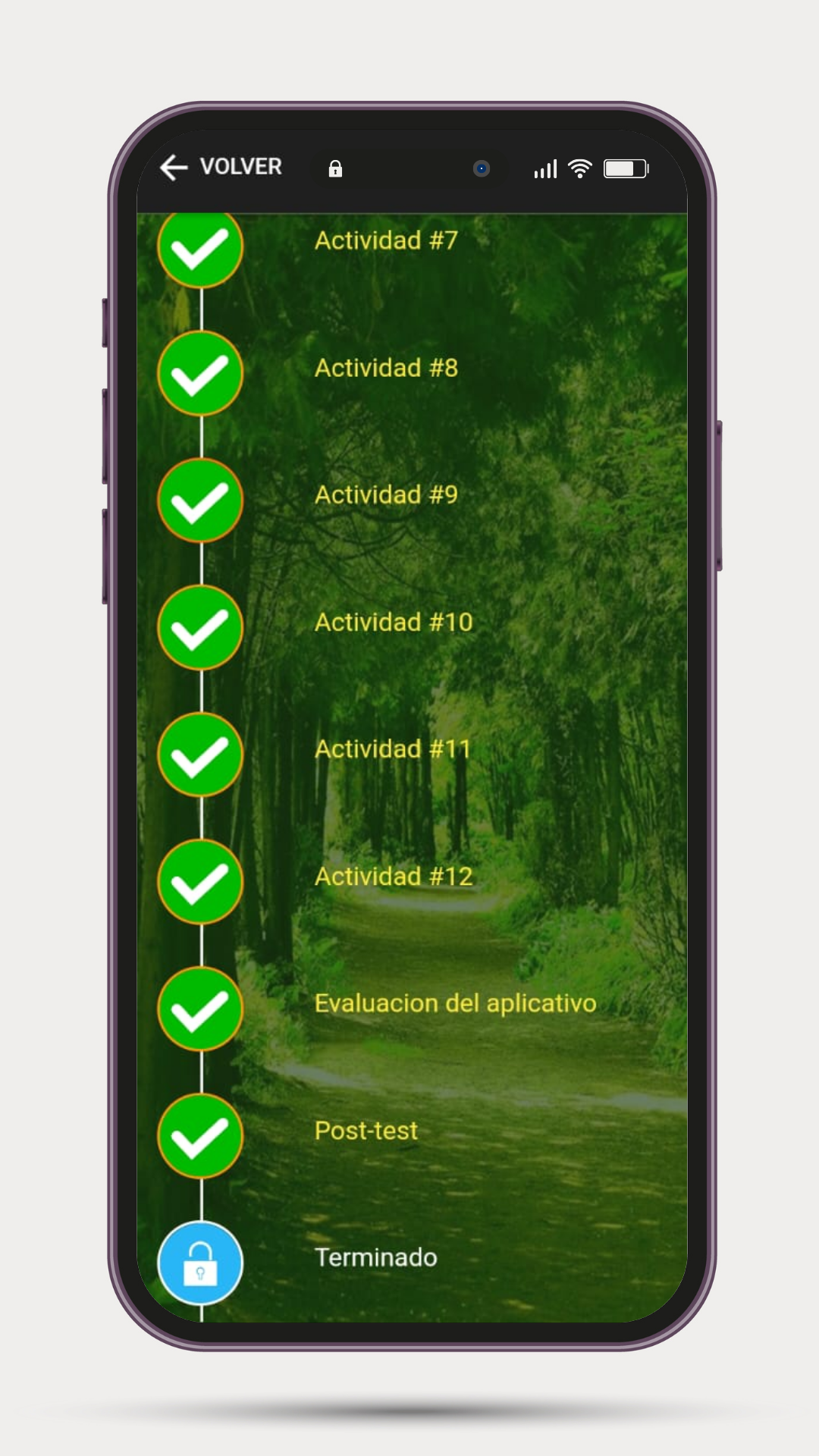


**Figure S4.**

*Recursos*


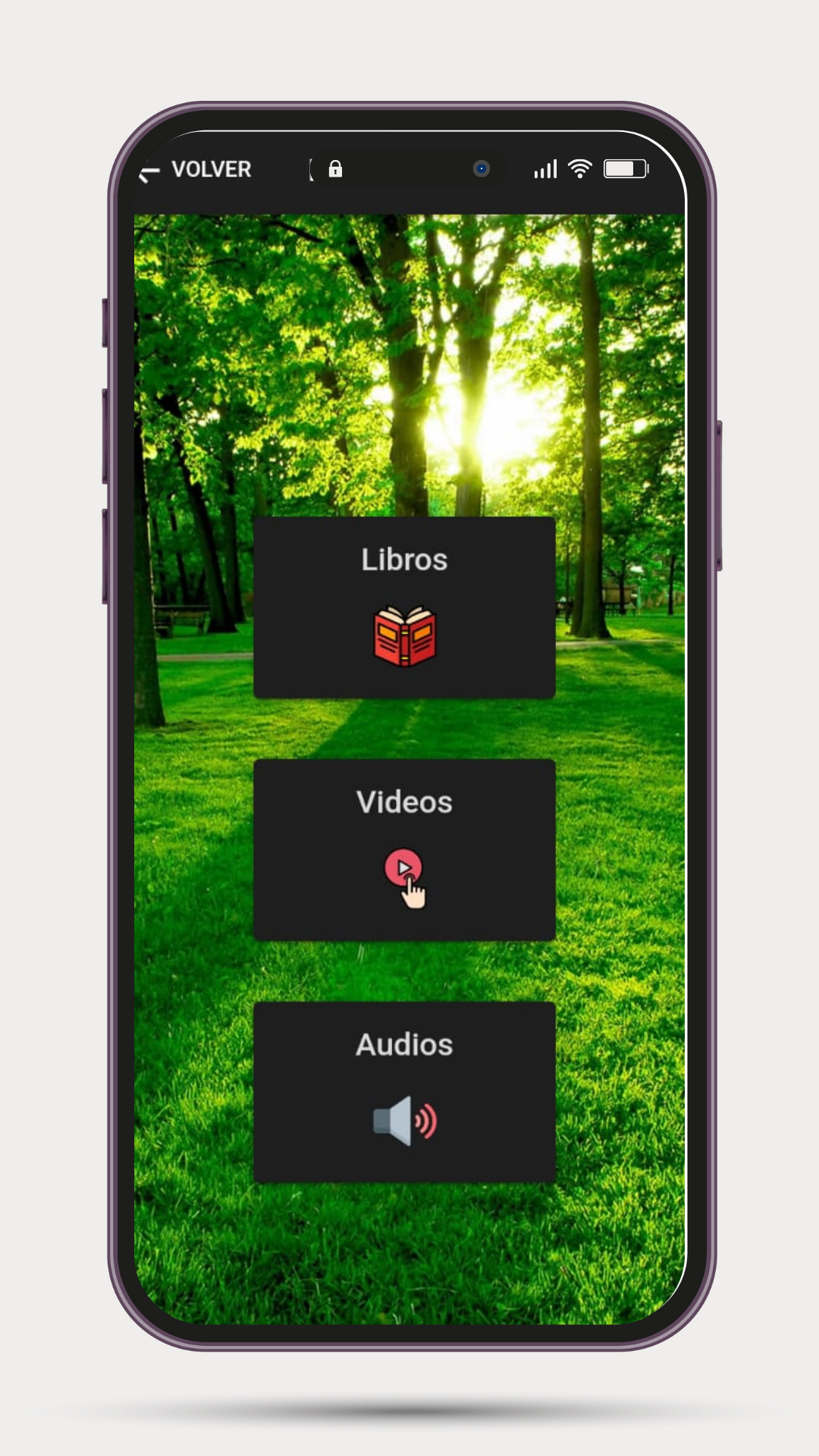


**Figure S5.**

*Informed Consent*


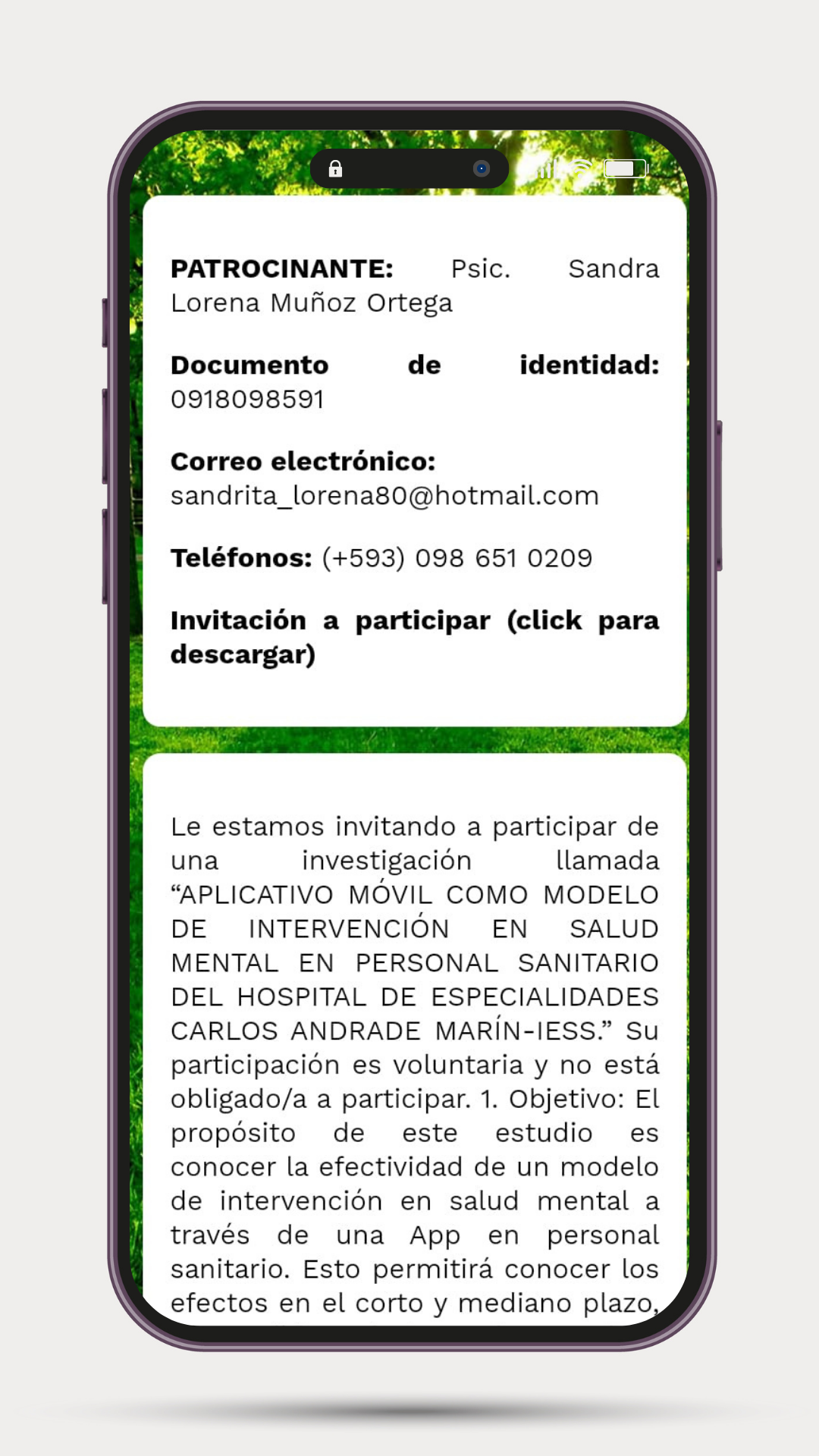


**Figure S6.**

*Help desk*


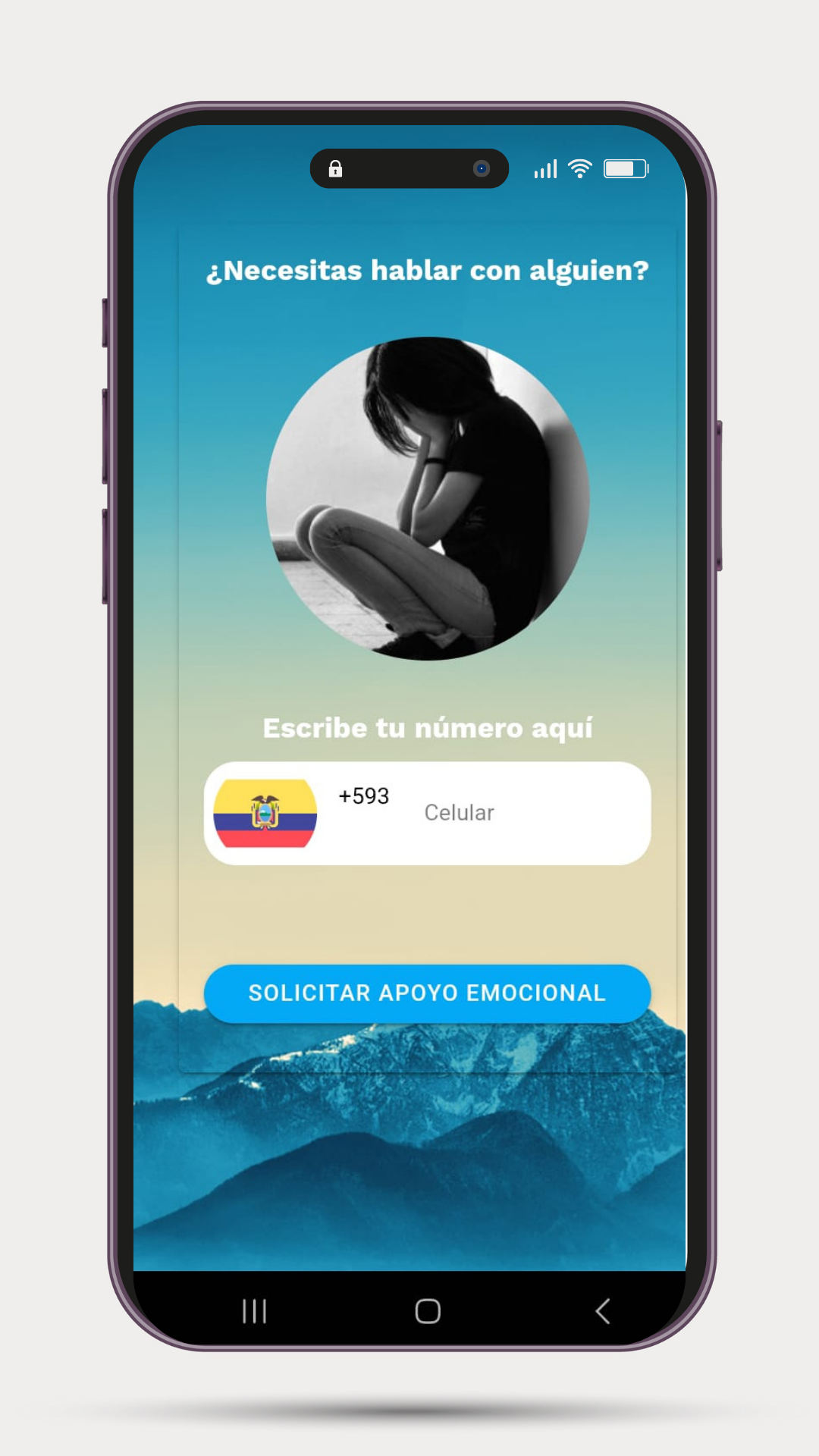


**Section S1: Informed Consent**

**English version**

**Informed Consent**

**SPONSOR:** Psic. Sandra Lorena Muñoz Ortega

**ID Document**: 0918098591

**Email:** sandrita_lorena80@hotmail.com

**Phone Number:** (+593) 098 651 0209

Invitation to Participate: We invite you to participate in a study titled **“MOBILE APPLICATION AS A MENTAL HEALTH INTERVENTION MODEL FOR HEALTHCARE STAFF AT CARLOS ANDRADE MARÍN SPECIALTY HOSPITAL-IESS.”**

Your participation is voluntary, and you are not obligated to participate.

1. **Objective:** The purpose of this study is to assess the effectiveness of a mental health intervention model through an app for healthcare personnel. This will allow us to understand the short- and medium-term effects, possible outcomes, and their magnitude, as well as protective factors. This knowledge will help us develop evidence-based recommendations and design future interventions to support your colleagues in dealing with mental health-related challenges. We aim to use the app with workers from various areas of the hospital, and your participation is highly valued given that you work in a hospital setting and experience that environment firsthand.
2. **Procedures and Duration:** If you agree to participate in this study, you will complete a two-stage survey. The survey is fully self-administered, confidential, and conducted online. In the first stage, you will answer general questions, as well as complete the PHQ-9 (9 questions) and GHQ-12 (12 questions) to assess your current mental health status. In the second stage, you will complete 12 mental health-promoting activities, each lasting 10 minutes, at your own convenience without specific time requirements. The only guideline is to complete at least one activity per week, allowing you to finish within three months. After three months, you will be asked to log back into the app and complete the PHQ-9 and GHQ-12 again to compare your results from before and after using the app. This will enable us to monitor your experiences and emotional state over time and possibly identify the onset of any mental health concerns.
3. **Risks:** While using the app, some questions may make you feel uncomfortable or emotionally affected. You may choose not to answer certain questions or even stop using the app at any time.
4. **Costs:** There is no financial cost for participating in this study.
5. **Benefits:** By using the app, you will have access to personal exercises that help you focus on aspects of your mental health. Additionally, your participation will contribute to validating technological tools for promoting mental health in healthcare workers, helping us offer improved health services.
6. **Compensation:** You will not receive financial compensation for participating.
7. **Confidentiality**: All information derived from your participation in this study will be kept strictly confidential on a secure server managed by the researcher. Data will be stored with password protection, accessible only to the researcher. Any identifying information will be modified to maintain anonymity. We guarantee that all working material and any scientific publications or communications will be entirely anonymous. Your private information will not be used for this research, nor will it be shared with other researchers for future studies.
8. **Voluntariness:** Your participation in this study is entirely voluntary, and you may withdraw from the study at any time. Declining or stopping participation in the study will not affect your employment status, result in reprimands from your supervisor, or create any discomfort related to your work position.
9. **Anonymization:** Participant anonymity will be maintained by generating a code upon entering the app, so personal data will not be linked to participants.
10. **Alternatives:** We do not have an alternative means of participation if you decide not to participate in this study.

**Future Interventions:** Periodic proposals to enhance healthcare workers' mental health may be developed based on this initial intervention.

**Participant Rights:** You may save a complete PDF copy of this document by clicking “Accept” on your screen or by signing the printed document. If you need further information about your participation in this study or believe you have been affected by it, you may contact the Principal Investigator, Psic. Sandra Lorena Muñoz Ortega, to resolve the issue and determine how best to assist you. For questions about your rights, you should contact the President of the “Human Research Ethics Committee” at IESS in Quito.

**Conclusion:** After having received and understood the information in this document and clarifying all my doubts, I give my consent to participate in the project “MOBILE APPLICATION AS A MENTAL HEALTH INTERVENTION MODEL FOR HEALTHCARE STAFF AT CARLOS ANDRADE MARÍN SPECIALTY HOSPITAL-IESS.”

**Accept: ___ Reject: ___**

**Full Name: __________________________________________**

**ID Number: ___________________**

**Date: ______________**_____

**Spanish version**

**Consentimiento informado**

**PATROCINANTE:** Psic. Sandra Lorena Muñoz Ortega

**Documento de identidad:** 0918098591

**Correo electrónico:** sandrita_lorena80@hotmail.com

**Teléfono**s: (+593) 098 651 0209

Invitación a participar: Le estamos invitando a participar de una investigación llamada **“APLICATIVO MÓVIL COMO MODELO DE INTERVENCIÓN EN SALUD MENTAL EN PERSONAL SANITARIO DEL HOSPITAL DE ESPECIALIDADES CARLOS ANDRADE MARÍN-IESS.”**

Su participación es voluntaria y no está obligado/a participar.

1. **Objetivo:** El propósito de este estudio es conocer la efectividad de un modelo de intervención en salud mental a través de una App en personal sanitario. Esto permitirá conocer los efectos en el corto y mediano plazo, los posibles efectos y la magnitud de estos, además de los factores protectores. Este conocimiento nos va a ayudar a desarrollar recomendaciones basadas en evidencia y permitirá diseñar otras intervenciones para apoyar a sus colegas a enfrentar diferentes situaciones que estén relacionadas con la salud mental en el futuro. Queremos usar el aplicativo en trabajadores de diferentes áreas del hospital, es muy importante contar con su participación, dado que Ud. Está trabajando en un ambiente hospitalario y está viviendo la experiencia de ese ambiente laboral.
2. **Procedimientos y duración:** Si acepta participar en este estudio, responderá una encuesta en dos etapas. Esta encuesta es completamente auto-aplicada y confidencial y se hace de forma on-line. En la primera etapa, se preguntan datos generales, y se aplica el test PHQ-9 el cual consta de 9 preguntas y GHQ-12, el cual consta de 12 preguntas, que nos permitirá conocer su estado de salud mental en la actualidad. La segunda etapa usted deberá realizar 12 actividades para el fomento de su salud mental, las cuales tienen una duración de 10 minutos cada una, y las podrá realizar en el lugar y tiempo que usted estime conveniente, no hay horarios ni tiempos establecidos para la realización de los mismos, la única consigna es que se elabore al menos uno por semana, para que en el plazo de 3 meses se pueda culminar. En esta segunda etapa se le pedirá después de 3 meses que ingrese nuevamente al aplicativo y conteste nuevamente el test PHQ 9 y GHQ-12, con la finalidad de establecer una comparación de antes y después del uso del aplicativo. De esta forma, podremos hacer un seguimiento de sus experiencias y estado emocional a lo largo del tiempo y posiblemente detectar la aparición de un problema de salud mental.
3. **Riesgos:** Durante el desarrollo del aplicativo, tal vez pueda sentirse incómodo/a o emocionalmente afectado/a por algunas de las preguntas. En cualquier momento puede decidir no contestar ciertas preguntas o incluso abandonar el uso del aplicativo en el momento que lo desee.
4. **Costos:** Su participación en este estudio no tendrá ningún costo económico para usted.
5. **Beneficios:** Al usar el aplicativo obtendrá el acceso a tareas personales que le ayudarán a enfocarse en aspectos de su salud mental. Además, su participación en este estudio contribuirá al validar herramientas tecnológicas para el fomento de la salud mental en trabajadores sanitarios que nos permitan brindar una mejor atención en salud.
6. **Compensación:** Usted no recibirá compensación económica por su participación.
7. **Confidencialidad:** Toda la información derivada de su participación en este estudio, será conservada bajo estricta confidencialidad, en un servidor seguro del investigador. Estos datos serán almacenados en forma protegida a través de una contraseña, al cual sólo tiene acceso el investigador. De igual forma, se modificará cualquier otra información que permita conocer su identidad. Se garantiza que todo el material de trabajo, así como cualquier publicación o comunicación científica, será completamente anónima. Su información privada no será utilizada para esta investigación, ni tampoco se la entregaremos a otros investigadores para el desarrollo de futuras investigaciones.
8. **Voluntariedad**: Su participación en este estudio es totalmente voluntaria y usted se puede retirar del estudio en el momento que lo desee. La no participación o interrupción de la participación en el estudio, no generará desvinculación de su puesto laboral, ni llamados de atención de su jefe inmediato superior, ni ninguna situación que genere malestar a su situación laboral.
9. **Anonimización:** Se mantendrá el anonimato del participante, generando un código al momento de su ingreso al aplicativo, por lo que no se conocerá los datos personales de los participantes.
10. **Alternativas:** No tenemos una forma alternativa de participar para ofrecerle, si decide no participar en este estudio.
11. **Futuras intervenciones:** Se plantea generar propuestas periódicas para incrementar la salud mental del personal sanitario, basada esta primera intervención.

**Derechos del participante:** Ud puede guardar una copia íntegra en pdf, de este documento una vez haga “click” en el botón “Acepto” que ve en estos momentos en su pantalla, o firmar el documento impreso. Si requiere cualquier otra información sobre su participación en este estudio, o cree que ha sido perjudicado al participar, puede comunicarse con la Investigadora Principal, Psic. Sandra Lorena Muñoz Ortega, para resolver el problema e identificar la forma de poder ayudarle. En caso de duda sobre sus derechos, debe comunicarse con el Presidente del “Comité de Ética de Investigación en Seres Humanos”, del IESS de la ciudad de Quito.

**Conclusión:** Después de haber recibido y comprendido la información de este documento y de haber podido aclarar todas mis dudas, otorgo mi consentimiento para participar en el proyecto “APLICATIVO MÓVIL COMO MODELO DE INTERVENCIÓN EN SALUD MENTAL EN PERSONAL SANITARIO DEL HOSPITAL DE ESPECIALIDADES CARLOS ANDRADE MARÍN-IESS”.

Acepto: ___ Rechazo: ___

Nombres y apellidos:__________________________________________

Cédula de identidad:___________________ Fecha;___________________________

**Section S2.** User Evaluation

1. **What did you think of the app?**

- Bad
- Fair
- Good
- Very good

1. **Was it easy to use?**

- Yes
- No

1. **Do you feel it helped improve your mental health?**

- Yes
- No

1. **Would you recommend the app to others?**

- Yes
- No

1. **If you faced a situation affecting your mental health, would you use the app again?**

- Yes
- No

1. **Did you like the app's presentation?**

- Yes
- No

1. **How has your mood and distress management improved after using the app for 12 weeks?**

- Your mood has worsened
- Your mood has not improved
- Your mood has improved somewhat
- Your mood has greatly improved
